# Mitochondrial Control of Myelination, Bioenergetics, Oxidative Stress, and the Pathogenesis of Optic Neuropathies

**DOI:** 10.3390/ijms27177678

**Published:** 2026-08-27

**Authors:** Haya Sahibzada, Rizwan Malik, Khaled K. Abu-Amero

**Affiliations:** Research Department, King Khaled Eye Specialist Hospital and Research Center, Riyadh 11462, Saudi Arabia

**Keywords:** myelination, mitochondria, oxidative stress, optic neuropathy, retinal ganglion cell, *OPA1*, bioenergetics, demyelination

## Abstract

Myelination, mitochondrial bioenergetics, and oxidative stress are usually discussed as separate problems in optic nerve disease. This review draws them together and reads the published evidence through a single variable, the balance between the energy a retinal ganglion cell (RGC) axon spends and the energy its mitochondria can supply. We review the role of myelin in conduction and axonal support, the mitochondrial cost of building and maintaining it, the vulnerability of oligodendrocytes and myelin to oxidative injury, and the nuclear control of mitochondrial output. We summarize the inherited optic atrophies linked to *OPA1*, *OPA3*, *AFG3L2*, *SPG7*, and *TMEM126A*, and set these primary mitochondrial disorders against the immune-mediated demyelinating optic neuropathies. Published studies already support several parts of this picture, including the energetic cost of demyelination, the mitochondrial dependence of RGC axons, and oxidative injury in inflammatory lesions. Drawing on that evidence, we propose, as a testable hypothesis rather than a settled mechanism, that optic nerve degeneration is favored when axonal ATP demand outruns mitochondrial supply, most sharply where the axon crosses from its unmyelinated to its myelinated segment near the lamina cribrosa. We use this framework to separate initiating lesions from disease modifiers and downstream consequences, and to set out therapeutic predictions open to experimental and clinical tests.

## 1. Introduction

The eye is among the first organs to reveal a mitochondrial deficit, because retinal ganglion cells and photoreceptors run some of the highest metabolic rates in the body. This review connects seven strands that are usually treated on their own: the function of myelin, the mitochondrial cost of making and running it, the effect of oxidative stress on oligodendrocytes and myelin, the nuclear control of mitochondrial biogenesis, the nuclear gene defects behind inherited optic atrophy, immune-driven demyelination, and the distribution of mitochondria across ocular tissue. Read apart they look like seven topics. Read together they track one variable, the balance between the energy a retinal ganglion cell axon needs and the energy its mitochondria can deliver [1,2]. Myelin is the demand side of that balance, mitochondrial and nuclear function the supply side, and oxidative stress the accelerant that damages both.

The central claim of this review is that the named optic neuropathies are not separate diseases with separate mechanisms but rather different entry points into the same energetic failure, and that the retinal ganglion cell axon at the lamina cribrosa is where that failure first becomes visible [3]. We advance this as a hypothesis rather than a settled conclusion, and it will need direct testing in future clinical studies designed with it in mind.

## 2. Myelination, Mitochondrial Bioenergetics, and Oxidative Stress

### 2.1. Myelin and Axonal Function

Myelination is the process in which oligodendrocytes in the central nervous system and Schwann cells in the peripheral nervous system wrap axons in multiple spiral layers of lipid-rich myelin. The sheath acts as an electrical insulator that raises membrane resistance and lowers membrane capacitance. It also organizes specialized axonal domains such as the nodes of Ranvier and the paranodal regions, which reduce current leakage and enable saltatory conduction, so action potentials jump between nodes and travel quickly while using little energy [1,4].

Myelin does more than insulate. Myelinating glia gives metabolic support to the axons they ensheath, delivering the energy substrates that sustain neuronal activity, a coupling that matters most for long axons with high energy budgets [5]. Myelin also protects the long-term structural integrity of the axon, and loss of myelin can drive axonal degeneration even when the neuron survives [6]. Recent work adds a role in neural plasticity and circuit tuning. Myelination therefore supports not only fast conduction but the survival and durability of the axon itself. These electrical and metabolic functions make myelin inseparable from axonal bioenergetics, because changes in sheath integrity alter both the energetic burden on the axon and the metabolic support provided by myelinating glia.

### 2.2. Mitochondrial Demands of Myelination

The same sheath that reduces the energetic cost of impulse propagation is itself metabolically expensive to build and maintain. Myelination depends on efficient mitochondrial function in oligodendrocytes and Schwann cells. During myelin formation, these glial cells expand membranes and synthesize large amounts of protein and lipid, requiring sustained ATP production through oxidative phosphorylation [7]. Beyond ATP production, mitochondrial metabolism supplies carbon substrates for myelin lipid synthesis. Glucose-derived pyruvate enters the tricarboxylic acid cycle and generates citrate, which is exported to the cytosol and converted to acetyl-CoA, a precursor for fatty acids and cholesterol.

Because roughly 70 to 80 percent of myelin is lipid, continuous fatty acid and cholesterol synthesis is needed for both formation and maintenance, and growth factors such as insulin-like growth factor 1 push lipid biosynthesis during active myelination [8,9]. As oligodendrocytes mature, they also feed the axon, converting glucose to lactate and exporting it through monocarboxylate transporters to axonal mitochondria for ATP [5,10]. Mitochondrial dysfunction therefore cuts ATP, lowers lipid synthesis, disrupts axon–glia metabolic support, and drives defective myelination, myelin loss, and axonal damage.

### 2.3. Oxidative Stress and Myelin Integrity

Because myelin formation and maintenance impose substantial metabolic demands, oxidative injury can disrupt both the glial cell and the sheath it produces. Oxidative stress impairs oligodendrocyte survival, differentiation, and function. These cells are particularly vulnerable because they combine high metabolic demand, abundant iron, and a heavy lipid load with comparatively limited antioxidant defenses [11,12]. Excess reactive oxygen species can cause mitochondrial dysfunction, DNA damage, protein oxidation, and lipid peroxidation. Since myelin is predominantly lipid, membrane lipid oxidation can directly compromise sheath integrity.

Reactive oxygen species also block the differentiation of oligodendrocyte precursor cells into mature myelinating cells, which lowers both developmental myelination and repair after injury [11,13]. Experimental oxidant exposure suppresses myelin-associated genes and arrests oligodendrocyte maturation, and it depletes the ATP needed for lipid synthesis and membrane expansion [14]. Chronic oxidative injury drives oligodendrocyte apoptosis and feeds neuroinflammation that worsens demyelination, a pattern seen in multiple sclerosis and related disorders [15]. Across these routes, oxidative stress compromises both myelin integrity and long-term axonal health.

## 3. Nuclear Control of Mitochondrial Function in Oligodendrocytes

Oligodendrocyte development and myelination depend on bidirectional communication between the nuclear and mitochondrial genomes. Nuclear-encoded transcriptional regulators coordinate mitochondrial metabolism, ATP output, calcium signaling, and lipid synthesis, all of which are required for the transition from an oligodendrocyte precursor cell to a mature myelinating cell [16].

Peroxisome proliferator-activated receptor-γ (PPAR-γ) drives oligodendrocyte maturation through mitochondria. Its activation raises myelin basic protein expression, extends oligodendrocyte processes, and increases respiratory chain complex IV activity without changing membrane potential, which improves ATP availability and supports the oscillatory calcium signaling that differentiation needs. Blocking mitochondrial respiration with rotenone abolishes those calcium oscillations and impairs myelination, and PPAR-γ agonists restore both, which shows that nuclear control of mitochondria feeds directly into myelin formation [16]. Downstream, PGC-1α promotes mitochondrial biogenesis and respiratory capacity to meet the demand of active myelin synthesis, so developing oligodendrocytes carry many elongated, high-output mitochondria [17].

After myelination, cellular metabolism shifts. Mitochondrial density falls, the network becomes more fragmented, oxidative phosphorylation decreases, and mature oligodendrocytes rely more heavily on glycolysis, exporting lactate through monocarboxylate transporter 1 to support axonal metabolism [5,18]. Experimental work supports the importance of mitochondrial respiration, calcium signaling, and oligodendrocyte-derived metabolic support for myelination and axonal maintenance. However, the broader proposition that disruption of mito-nuclear communication is a common causal pathway across optic neuropathies remains an interpretation of convergent evidence rather than a mechanism demonstrated in every disease context.

## 4. Nuclear Gene Defects and Inherited Optic Atrophy

Autosomal dominant optic atrophy (ADOA) and autosomal recessive optic atrophy (AROA) are best understood as mitochondrial optic neuropathies driven by nuclear genes that govern mitochondrial structure, protein quality control, and energy balance. Retinal ganglion cells carry no mtDNA mutation in these disorders, yet they are acutely sensitive to defects in nuclear-encoded mitochondrial proteins that control dynamics, inner membrane organization, and cristae maintenance. The shared pathology is disturbed fusion and fission balance, weakened oxidative phosphorylation, altered apoptotic signaling, and failure of inner membrane upkeep [19].

*OPA1* is the central gene in ADOA. It encodes a dynamin-related GTPase in the inner membrane and intermembrane space that drives inner membrane fusion, maintains cristae, preserves mtDNA, and restrains apoptosis. Loss of *OPA1* fragments the network, disorganizes cristae, lowers membrane potential, impairs respiration, and makes cytochrome c release and apoptosis more likely [19]. Disease arises through haploinsufficiency from truncating alleles and, for some missense alleles in the GTPase domain, through dominant negative interference with fusion [20]. The result is a fragmented, bioenergetically weak mitochondrial population in a cell type that depends heavily on mitochondrial distribution and oxidative phosphorylation, which explains the selective loss of retinal ganglion cells.

Mutations in *OPA3* cause both dominant and recessive optic atrophy. The recessive form, Costeff syndrome, combines early bilateral optic atrophy with later spasticity and extrapyramidal signs. The disease-relevant transcript, *OPA3A*, localizes to mitochondria, and elevated urinary 3-methylglutaconic and 3-methylglutaric acids are consistent with a mitochondrial metabolic defect [21].

Across these genes, the routes differ, but the endpoint is shared. Whether the lesion is direct, as in *OPA1*, or indirect, as in *OPA3*, *AFG3L2*, and *SPG7*, the downstream picture converges on mitochondrial fragmentation, cristae remodeling, reduced ATP, higher oxidative stress, and a lowered apoptotic threshold, with retinal ganglion cells as the most vulnerable target.

## 5. Reactive Oxygen Species and Immune-Mediated Demyelination

Reactive oxygen species drive immune-mediated demyelination by generating oxidative stress during neuroinflammation. Antioxidant systems normally hold them in check, but activated immune cells overproduce reactive oxygen species and overwhelm those defenses, damaging oligodendrocytes and myelin and pushing the disease forward [22].

The central nervous system is especially exposed, because of high oxygen use, lipid-rich myelin, and the weak antioxidant capacity of oligodendrocytes. Myelin membranes, with their high lipid to protein ratio, are prime targets for oxidative lipid damage, which weakens membrane integrity and makes the sheath easier to degrade [23]. Inflammatory lesions are packed with activated microglia and macrophages that generate superoxide through NADPH oxidase during the respiratory burst, and they sit against myelin sheaths, exposing them to high oxidant loads. Pro-inflammatory cytokines such as interferon-γ and interleukin 1 raise that output further [24]. Experimental autoimmune encephalomyelitis and multiple sclerosis show increased superoxide, hydrogen peroxide, lipid peroxidation products, and oxidative change inside plaques [25].

Oligodendrocytes are hit hardest because, compared with astrocytes, they carry much lower glutathione, weaker glutathione peroxidase, no manganese superoxide dismutase, and no metallothionein. Reactive oxygen species generated in vitro selectively kill oligodendrocytes while sparing astrocytes and macrophages [26]. Reactive oxygen species also damage myelin directly through lipid peroxidation and oxidation of myelin basic protein and proteolipid protein, and they activate matrix metalloproteinases that degrade myelin basic protein, which speeds demyelination. Repair suffers too, because remyelination proceeds in an oxidative field that harms remyelinating oligodendrocytes and leaves the job incomplete [27]. Antioxidant treatments, including catalase, superoxide dismutase, and synthetic scavengers, reduce disease severity in these models, which points to oxidative control as a plausible therapeutic route [22].

## 6. Distribution of Mitochondria in Ocular Tissue

**Figure 1:**
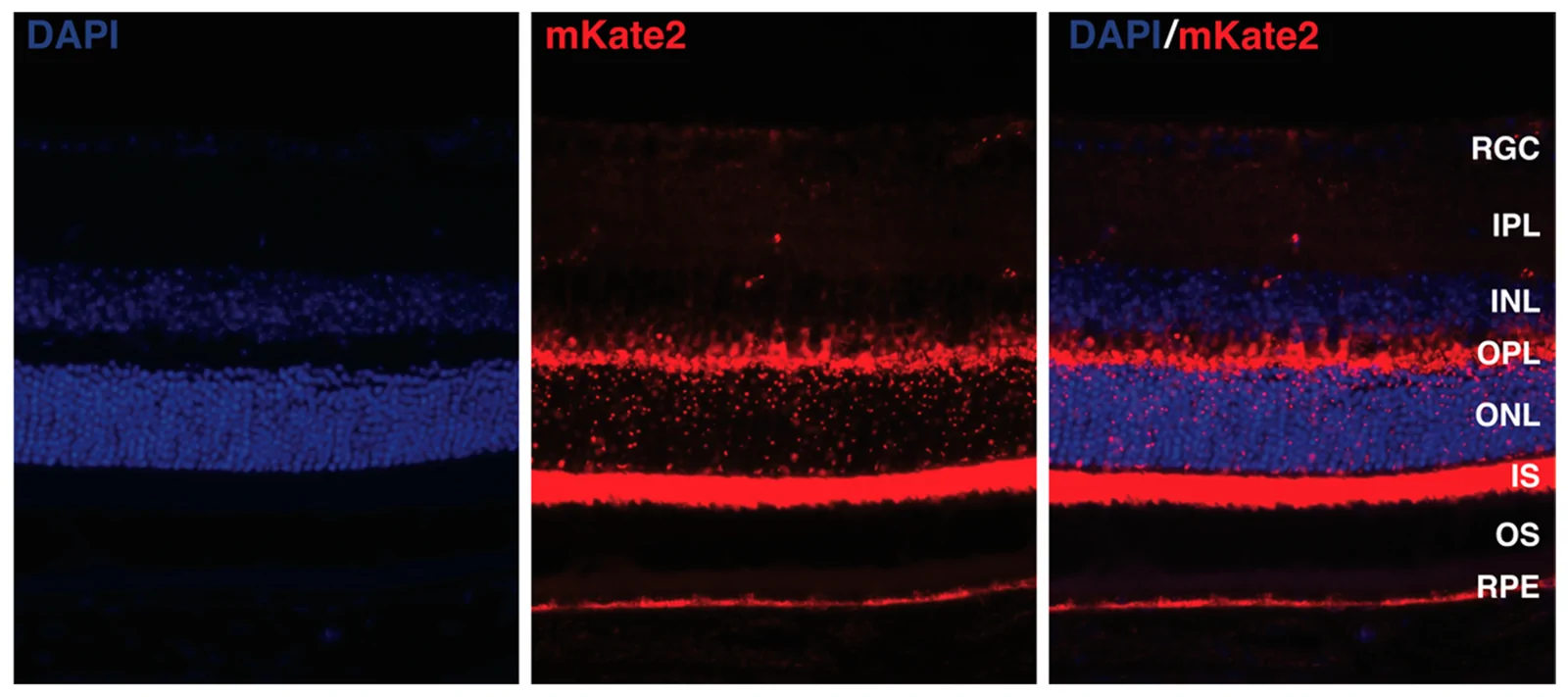
Representative fluorescence micrographs of a mouse retina in cross section. Nuclei stained with DAPI are shown in blue, mitochondria expressing the mito::mKate2 reporter are shown in red, and the merged image is shown in the right panel. The mitochondrial signal is most prominent in photoreceptor inner segments, with additional signal in the plexiform and ganglion cell regions. Abbreviations: RGC, retinal ganglion cell; IPL, inner plexiform layer; INL, inner nuclear layer; OPL, outer plexiform layer; ONL, outer nuclear layer; IS, inner segment; OS, outer segment; RPE, retinal pigment epithelium.

Mitochondria are distributed across ocular tissues according to local metabolic requirements, with high densities in compartments that require continuous ATP production for phototransduction, ion transport, and neuronal signaling [2]. Figure 1 provides a histological example of this principle in the retina: the mito::mKate2 signal is especially prominent in photoreceptor inner segments, with additional mitochondrial signal in plexiform layers and the ganglion cell region. Retinal pigment epithelial cells position mitochondria basally near Bruch membrane and the choriocapillaris, supporting transport, outer-segment phagocytosis, and barrier function. The inner retina is also mitochondria-rich, particularly in RGCs and the nerve fiber layer [2].

Retinal ganglion cells show a distinctive pattern. Mitochondria cluster in the perinuclear cytoplasm, along dendrites in the inner plexiform layer, and inside the unmyelinated axons of the nerve fiber layer, where they accumulate in regularly spaced varicosities that sustain axonal transport and action potential propagation. Because these axons run long distances into the optic nerve, mitochondrial transport is essential for survival [3].

**Figure 2:**
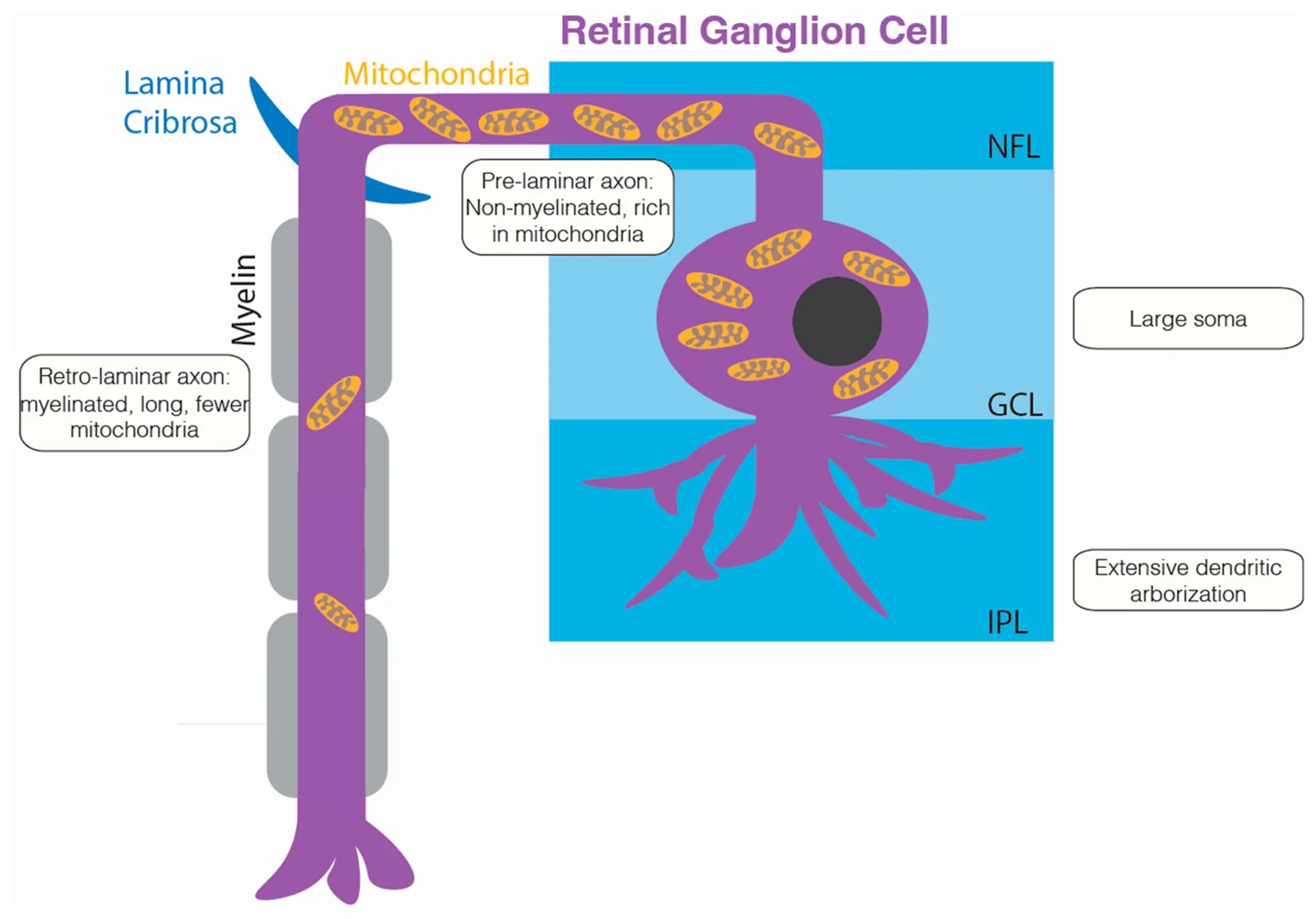
The cytoarchitecture of retinal ganglion cells and its high metabolic demand. Retinal ganglion cells have long axons and high ATP requirements to sustain continuous visual signal transmission. Mitochondria concentrate in the unmyelinated prelaminar axons, where energy demand is greatest, while the myelinated retrolaminar axons carry fewer mitochondria because saltatory conduction is more efficient. Abbreviations: IPL inner plexiform layer, GCL ganglion cell layer, NFL nerve fiber layer.

The optic nerve head is among the most metabolically demanding regions of the visual pathway. As illustrated schematically in Figure 2, mitochondria concentrate in the unmyelinated prelaminar axon and become less dense after the axon enters the myelinated retrolaminar nerve, where saltatory conduction lowers the energetic cost of impulse propagation [3].

Other ocular tissues follow the same logic. Corneal endothelial cells hold abundant mitochondria to power the Na+/K+-ATPase that maintains deturgescence and transparency, and their dysfunction drives Fuchs endothelial corneal dystrophy [28]. The non-pigmented ciliary epithelium carries the mitochondria that fuel aqueous humor secretion [29]. In the lens, mitochondria are confined to anterior epithelial and superficial fiber cells and are cleared as fiber cells enter the organelle-free zone, which keeps the lens transparent [30]. Extraocular muscles hold one of the highest mitochondrial contents of any skeletal muscle, and their dysfunction underlies chronic progressive external ophthalmoplegia [31]. The placement of mitochondria across the eye also shapes vulnerability to light and to age-related stress [32,33,34].

## 7. Discussion

**Figure 3:**
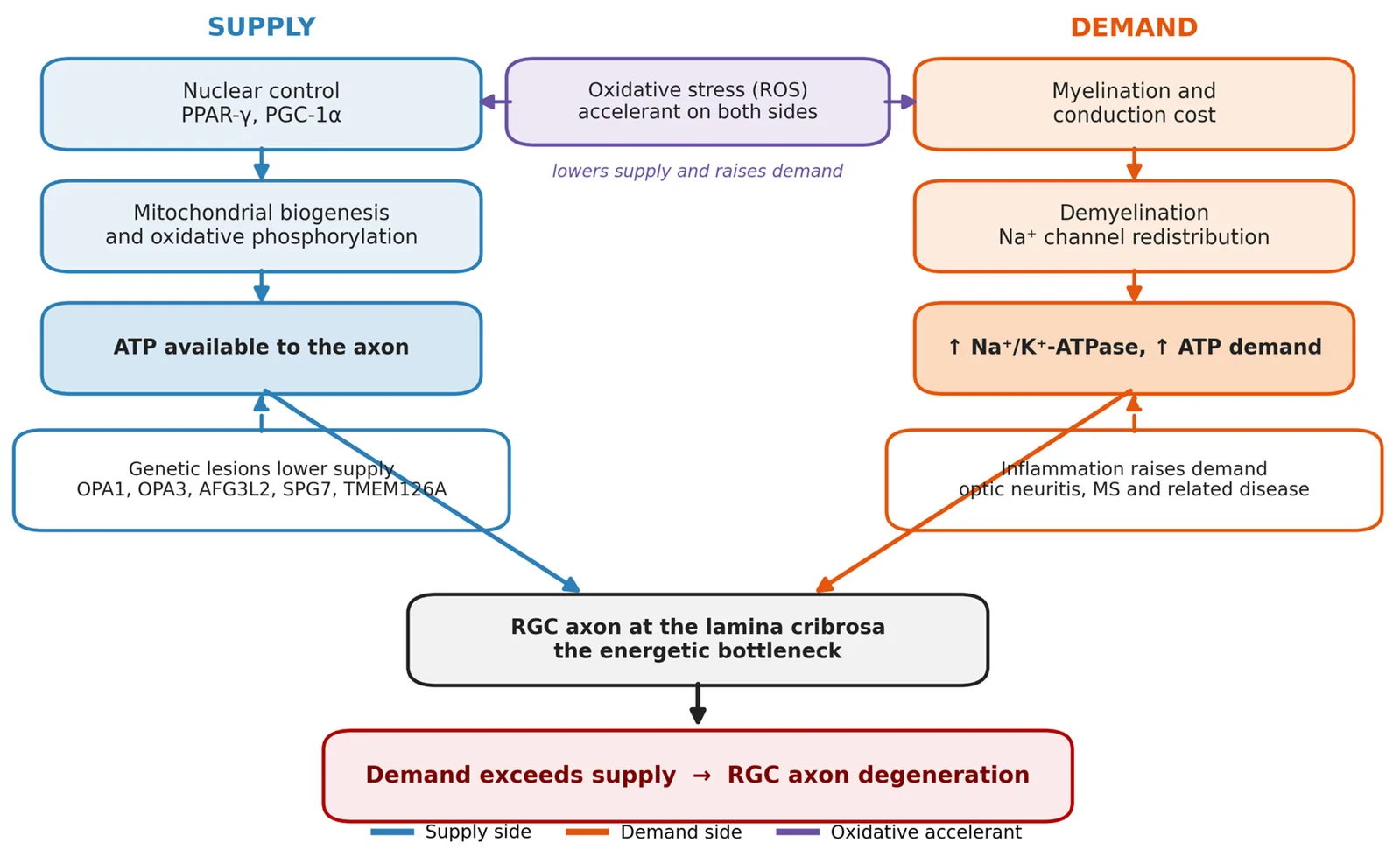
The bioenergetic convergence model proposed in this review. The model hypothesizes that optic nerve degeneration is favored when axonal ATP demand exceeds mitochondrial supply near the RGC axon as it crosses the lamina cribrosa. Nuclear control through PPAR-γ and PGC-1α supports mitochondrial biogenesis and oxidative phosphorylation, whereas genetic lesions in *OPA1*, *OPA3*, *AFG3L2*, *SPG7*, and *TMEM126A* can reduce mitochondrial reserve. Demyelination can increase ionic pumping requirements and therefore ATP demand, whereas oxidative stress may lower supply and, in inflammatory disease, worsen myelin injury. Table 1 distinguishes disorders in which these processes are initiating events from those in which they are modifiers or downstream consequences.

The preceding sections describe myelin, its mitochondrial cost, the oxidative stress that attacks it, the nuclear control of mitochondrial output, the nuclear gene defects behind inherited optic atrophy, immune-driven demyelination, and the ocular distribution of mitochondria. This discussion argues that they are one story, and that the balance between axonal energy demand and mitochondrial energy supply is the disease (Figure 3).

### 7.1. The Central Hypothesis

The optic nerve degenerates whenever axonal energy demand exceeds mitochondrial energy supply, and the retinal ganglion cell fails first because its axon crosses an energetic discontinuity at the lamina cribrosa. Two quantities set that balance, and it is worth defining them at the outset. Supply is the rate at which the axon’s mitochondria regenerate ATP through oxidative phosphorylation. Demand is the rate at which the axon spends ATP to restore the ionic gradients that conduction dissipates, most of it consumed by the sodium potassium ATPase clearing the sodium that enters during firing. Both are rates of ATP turnover, and the axon degenerates when spending outruns production. Demyelination raises demand through a specific electrophysiological change. When the sheath is lost, the sodium channels normally clustered at the nodes of Ranvier redistribute along the bared internode, so sodium enters across the whole membrane rather than at the nodes alone, and the sodium potassium ATPase must hydrolyze more ATP to pump it back out [22]. Loss of saltatory conduction is therefore, in energetic terms, a rise in axonal demand rather than a change in a different kind. Two classes of insult push the system past threshold. Demyelination and inflammation raise demand. Mitochondrial dynamics defects, nuclear-encoded bioenergetic failure, and oxidative stress lower supply. Oxidative stress needs a more exact place in this scheme than one side of the balance allows, because it is at once a consequence of the imbalance and a cause of it. Mitochondria that are failing, whether from a dynamics defect or a nuclear lesion, leak reactive oxygen species, and those species then oxidize the respiratory chain and mitochondrial membranes and lower supply further, while also degrading myelin and blocking the maturation of its precursors, which raises demand. Oxidative stress therefore rarely starts the process. It accelerates whichever failure is already under way and closes a feed-forward loop that carries the axon past threshold faster than a pure loss of supply or a pure rise in demand would on its own. The named optic neuropathies are not separate diseases with separate mechanisms. They are different entry points into the same energetic failure [3,35].

### 7.2. Why the Retinal Ganglion Cell Fails First

The intraocular portion of the retinal ganglion cell axon carries no myelin, so it conducts continuously rather than by saltatory jumps. Continuous conduction opens sodium channels along the whole membrane, and the sodium–potassium ATPase then burns ATP over that entire length to restore the ion gradient. Mitochondria concentrate in this unmyelinated segment for that reason, and their density falls sharply once the axon acquires myelin behind the lamina cribrosa [3]. The axon meets its highest sustained energy load and its densest mitochondrial population in the same short stretch of tissue. Any drop in mitochondrial output strikes this segment before anywhere else, which is why the optic nerve head degenerates first in both hereditary and acquired optic neuropathy.

### 7.3. The Energetic Logic That Links the Sections

Building myelin is itself an energy sink. Oligodendrocytes and Schwann cells expand membrane and synthesize lipid on a large scale, and they draw the ATP, the acetyl-CoA, and the fatty acid precursors from mitochondrial metabolism, since roughly three quarters of myelin is lipid [5,8]. Oxidative stress attacks the same machinery, oxidizing membrane lipids and myelin proteins and arresting precursor maturation in cells with high iron, a heavy lipid load, and weak glutathione defenses [11]. Nuclear transcription sets the bioenergetic capacity of the cell, because PPAR-γ and PGC-1α govern mitochondrial biogenesis and oxidative phosphorylation capacity, so a nuclear lesion lowers the ATP that the cell can generate against a given demand rather than fixing an absolute upper limit [16]. Mature oligodendrocytes then feed the axon directly, exporting lactate through the monocarboxylate transporter to fuel axonal mitochondria [5,10]. These are not separate stories. They are the supply side of one equation.

### 7.4. Demyelination as Amplifier, Not Primary Cause

This relationship is a central interpretive step in the proposed framework. Removing myelin converts an efficient saltatory cable into a membrane that must support ionic flux over a larger surface area. Axonal energy demand rises as sodium channels redistribute and the Na+/K+-ATPase works harder to restore ionic gradients [22,24]. A healthy neuron may compensate for this additional load. An RGC with impaired mitochondrial capacity, whether from an *OPA1* or *AFG3L2* defect or from inflammatory mitochondrial injury, may not. In this setting, demyelination is clinically important not simply because myelin is absent, but because its loss increases an energetic burden that compromised mitochondria may be unable to meet. This interpretation also explains why demyelination cannot be the initiating lesion in inherited mitochondrial optic atrophies, which prominently affect the unmyelinated intraocular axon.

The mechanistic status differs across disease categories. In inherited *OPA1*-, *OPA3*-, *AFG3L2*-, *SPG7*-, and *TMEM126A*-associated optic atrophies, mitochondrial dysfunction in RGCs and their axons is considered a primary event, whereas a primary myelin lesion has not been established. In multiple sclerosis-associated optic neuritis and MOG antibody-associated disease, inflammatory demyelination is an initiating event, and mitochondrial dysfunction can act as a secondary amplifier of axonal injury. In AQP4-IgG-positive neuromyelitis optica spectrum disorder, astrocyte-directed autoimmunity is primary, with demyelination, oxidative injury, and axonal mitochondrial stress occurring downstream. The demand-supply model therefore describes a possible point of convergence, not a claim that all diseases share the same initiating mechanism.

### 7.5. Convergence Across Disease Categories

The inherited mitochondrial optic atrophies, including *OPA1*-associated dominant optic atrophy and optic atrophy associated with *OPA3*, *AFG3L2*, *SPG7*, and *TMEM126A*, are best classified as primary supply-side disorders because their initiating molecular defects directly perturb mitochondrial structure, quality control, or respiratory competence [19,20,21,36,37,38]. By contrast, multiple sclerosis-associated optic neuritis is initiated by immune-mediated inflammatory demyelination; mitochondrial dysfunction and oxidative injury are well-supported downstream contributors that can amplify axonal loss [22,23,24,25,26,27]. AQP4-IgG-positive NMOSD is primarily an astrocytopathy, while MOGAD is a primary inflammatory demyelinating disorder. In both, oxidative and mitochondrial abnormalities should therefore be interpreted as modifiers or consequences unless disease-specific evidence demonstrates a primary mitochondrial lesion [39,40]. These distinctions prevent the convergence model from conflating shared downstream bioenergetic stress with a shared cause.

### 7.6. Predictions That Follow

The relationships set out in the preceding sections are drawn from published evidence. What follows is not. These are predictions generated by the model, not results already established across optic neuropathies, and they are offered as hypotheses open to test. Three consequences should be observable if the hypothesis holds. First, any manipulation that raises axonal energy demand, including experimental demyelination, should accelerate degeneration more severely in retinal ganglion cells carrying a mitochondrial dynamics mutation than in wild-type cells, and the gap should be widest in the prelaminar segment. Second, raising mitochondrial supply, through PGC-1α activation, improved oxidative phosphorylation, or lactate delivery from oligodendrocytes by way of the monocarboxylate transporter, should protect axons against a demyelinating insult without restoring myelin [10]. Third, antioxidant defense and remyelination therapy should act together rather than independently, because one protects supply while the other lowers demand. Each prediction is testable in existing rodent and cell models, and each would falsify the hypothesis if it failed. Until they are tested directly, these predictions remain the inferential step of this review and should be read as separate from the mechanisms it summarizes.

The mechanisms outlined above are not confined to the optic nerve. Each category of defect discussed in this review—structural myelin gene mutations, impaired mitochondrial or nuclear bioenergetic support, and oxidative or immune-mediated attack on myelin—has a recognized neurological disease counterpart, illustrating that the demand–supply framework developed here extends across the nervous system.

### 7.7. Structural and Compaction Defects of Myelin

Mutations in the genes that build and compact myelin produce a spectrum of peripheral and central hypomyelinating and demyelinating disorders. In the peripheral nervous system, duplication of PMP22 or point mutations in PMP22, MPZ, or the gap junction gene GJB1 (connexin 32) cause Charcot-Marie-Tooth disease type 1, the most common inherited demyelinating neuropathy, in which slowed nerve conduction and segmental demyelination reflect direct loss of the structural myelin proteins discussed in Section 2.1 [41,42]. In the central nervous system, mutations in PLP1, the major compact myelin proteolipid, cause Pelizaeus–Merzbacher disease and its milder allelic variant spastic paraplegia type 2, producing a spectrum from severe congenital hypomyelination to progressive spasticity later in life; mutations in the gap junction gene GJC2 produce a phenotypically similar autosomal recessive disorder, Pelizaeus–Merzbacher-like disease [43]. A related group of lysosomal and peroxisomal disorders, Krabbe disease (GALC), metachromatic leukodystrophy (ARSA), and X-linked adrenoleukodystrophy (ABCD1), do not mutate myelin structural proteins directly but instead disrupt the catabolism of myelin lipid components, so that toxic lipid accumulation or lipid depletion destroys myelin from within; these leukodystrophies illustrate that both the synthesis and the breakdown of myelin lipid—the same lipid-heavy composition discussed in Section 2.2—are points of vulnerability [44].

### 7.8. Mitochondrial and Nuclear-Encoded Bioenergetic Defects

A second group of diseases mirrors the supply-side mechanism developed in Section 3 and Section 4, in which nuclear genes governing mitochondrial replication, fusion, or protease activity fail to sustain the energetic demands of myelinating or myelinated cells, but with a neurological rather than an exclusively optic phenotype. Biallelic mutations in POLG, which encodes the mitochondrial DNA polymerase, cause a spectrum of disorders including Alpers-Huttenlocher syndrome and, when combined with thymidine phosphorylase (TYMP) loss in mitochondrial neurogastrointestinal encephalomyopathy, a true leukoencephalopathy; both reflect the same principle argued in Section 4, that a nuclear lesion constrains the ATP a cell can produce against demand, with the brain and its white matter among the first tissues to fail [45]. In the peripheral nervous system, mutations in MFN2, which encodes the mitochondrial fusion protein mitofusin 2, cause Charcot-Marie-Tooth disease type 2A, the most common axonal form of CMT; unlike CMT1, the pathology here is axonal rather than primarily demyelinating, underscoring that mitochondrial dynamics defects can manifest as either a demand-side myelin problem or a supply-side axonal problem depending on the cell type affected [46]. These disorders sit alongside the *OPA1*, *AFG3L2*, and *SPG7* mutations already discussed in Section 6: *OPA1* mutations can extend beyond isolated optic atrophy into ‘*OPA1* plus’ syndromes with sensorineural deafness, ataxia, and peripheral neuropathy, and *AFG3L2* and *SPG7* both also cause spinocerebellar ataxia and hereditary spastic paraplegia phenotypes when the same mitochondrial protease defect is more diffusely expressed.

### 7.9. Oxidative Stress and Immune-Mediated Demyelination

The immune-mediated mechanism described in Section 5 extends beyond multiple sclerosis to a family of related CNS demyelinating diseases that differ mainly in the antigen targeted and the pattern of injury. Neuromyelitis optica spectrum disorder is driven chiefly by antibodies against the astrocytic water channel aquaporin-4, producing severe, often bilateral optic neuritis and longitudinally extensive transverse myelitis with an injury pattern distinct from classical MS [39]. Myelin oligodendrocyte glycoprotein antibody-associated disease and acute disseminated encephalomyelitis represent further points on this spectrum, the former often relapsing and antibody-driven, the latter typically monophasic and post-infectious or post-vaccination; both converge, as MS does, on oligodendrocyte and myelin injury through reactive oxygen species and complement- or antibody-mediated mechanisms layered onto the oxidative vulnerability described in Section 2.3 [40]. A caution is needed here. These are primarily inflammatory and autoimmune diseases, and their initiating lesion is immune, not bioenergetic. Mitochondrial dysfunction and oxidative injury do appear in them, but as a downstream consequence of inflammation rather than as the primary cause. For that reason, they enter this review less as members of a shared pathogenetic mechanism and more as clinical mimickers within the differential diagnosis of a mitochondrial optic neuropathy, since an inflammatory optic neuritis and a hereditary optic atrophy can look alike at the optic nerve head even when their primary drivers differ.

Read together, these entities occupy the framework in different ways. The structural myelin gene defects, the lysosomal leukodystrophies, and the mitochondrial and nuclear bioenergetic disorders share a primary energetic logic, whether the lesion removes the sheath, blocks its synthesis, or starves it of ATP. The immune demyelinating diseases are placed alongside them with a clear qualification. Their primary mechanism is autoimmune, and they belong here mainly because their secondary oxidative and energetic injury can converge on the same optic nerve endpoint, which makes them important differential diagnoses rather than examples of the same primary cause. Which axis is affected, and in which cell type, still shape whether the phenotype is a peripheral neuropathy, a leukoencephalopathy, or an optic neuropathy.

### 7.10. Disease-Specific Evidence Classification

Table 1 summarizes the disease-specific evidence and explicitly distinguishes primary events, modifiers, and downstream consequences. Evidence strength is graded qualitatively as high, moderate, or limited according to the consistency and directness of human genetic, pathological, imaging, and experimental data cited in this review.

### 7.11. Clinical and Translational Perspectives

The model separates the optic neuropathies by where the energy balance fails, and that splits maps onto how patients present and how they are worked up. A painless, bilateral, slowly progressive optic atrophy with a family history points to a supply-side lesion and to genetic testing of *OPA1* and the wider panel of *OPA3*, *AFG3L2*, *SPG7*, and *TMEM126A*. A subacute, often painful, frequently unilateral optic neuritis with other central nervous system signs points to a demand-side inflammatory process and to imaging and antibody testing. The distinction matters in practice, because the two groups differ in prognosis and in treatment, and because the inherited mitochondrial optic neuropathies offer no immunosuppressive target [3,35].

The framework also suggests interventions more specific than nonspecific antioxidant or mitochondrial support. Supply-directed strategies include mutation-specific or gene-restoration approaches for nuclear mitochondrial disorders, preservation of mitochondrial fusion and cristae structure, enhancement of mitochondrial biogenesis, and support of axonal substrate delivery. Demand-directed strategies include active remyelination, promotion of oligodendrocyte precursor differentiation, stabilization of nodal and paranodal organization, and suppression of inflammatory mechanisms that increase ionic and metabolic load. A third category targets the coupling between the two sides, for example, by improving oligodendrocyte-to-axon lactate transfer or by combining remyelination with interventions that preserve mitochondrial reserve. These strategies follow directly from the model because they target identifiable nodes in the proposed pathway rather than oxidative stress in general [10,16,35].

These remain therapeutic hypotheses rather than established treatments for inherited mitochondrial optic atrophy. Importantly, the framework predicts that the most informative interventions will be those with measurable effects on either supply or demand, such as mitochondrial respiratory reserve, axonal ATP-sensitive physiology, visually evoked potential latency, or quantitative remyelination measures. It also predicts that combination approaches may outperform single-pathway therapy when mitochondrial reserve is low and myelin-related demand is simultaneously high.

### 7.12. Therapeutic Implications Beyond General Mitochondrial Support

The disease-specific classification in Table 1 suggests that therapy should be matched to the initiating lesion. In primary mitochondrial optic atrophies, rational approaches include gene replacement or gene editing where technically feasible, stabilization of mitochondrial dynamics and cristae architecture, and interventions that increase respiratory reserve before irreversible RGC loss. In inflammatory optic neuropathies, treatment of the immune trigger remains primary, but the framework suggests that remyelination and axonal metabolic protection could be layered onto immunotherapy after acute inflammation is controlled. The model therefore does not support a single antioxidant strategy across disorders; it predicts different combinations depending on whether the dominant defect lies in mitochondrial supply, myelin-related demand, or an upstream immune process.

### 7.13. Clinical Studies That Test Components of the Framework

Clinical evidence is still fragmentary, but several studies have tested components of the proposed model. In *OPA1*-dominant optic atrophy, a prospective phase 2 study of idebenone in 16 genetically confirmed patients reported small improvements or stabilization in visual outcomes over 12 months, although the investigators emphasized that the uncontrolled design could not separate treatment effects from placebo effects or natural history [47]. Thus, this study supports feasibility of supply-directed intervention but does not establish disease modification.

The strongest clinical test of a demand-directed strategy comes from multiple sclerosis. The ReBUILD randomized crossover trial enrolled patients with chronic demyelinating optic neuropathy and found that clemastine shortened visual-evoked potential latency, providing proof of principle that pharmacological remyelination can improve a physiological marker of optic pathway conduction [48]. Ongoing or more recent trials have extended this approach, including RESTORE, which evaluates clemastine with oculographic and visual outcomes, and CCMR Two, which tests metformin plus clemastine to promote remyelination in relapsing-remitting multiple sclerosis with chronic optic neuropathy [49,50]. These studies are relevant to the demand side of the framework, but they do not directly test whether remyelination and mitochondrial support act synergistically.

The clinical translation is also not uniformly positive. In the progressive multiple sclerosis TRAP-MS platform study, a clemastine arm was stopped early after safety criteria were triggered and accelerated disability progression was observed in the treated group [51]. This finding argues against assuming that a mechanistically attractive remyelinating intervention will be beneficial in every disease stage. Future trials that directly test the present framework should stratify patients by the initiating lesion and mitochondrial reserve, include biomarkers of both remyelination and mitochondrial function, and prospectively evaluate combination therapies rather than extrapolating from either pathway alone.

## 8. Conclusions

This review integrates established evidence on myelin biology, mitochondrial bioenergetics, oxidative injury, and optic neuropathy, while separating those findings from the unifying interpretation proposed here. The evidence supports primary mitochondrial dysfunction in inherited optic atrophies and primary inflammatory or astrocytic injury in immune-mediated optic neuropathies; it does not support a single initiating mechanism across these disorders. We therefore propose the demand–supply model as a testable framework for understanding how distinct upstream lesions may converge on RGC axonal energetic failure. Its main predictions are that disease severity will depend on mitochondrial reserve relative to axonal demand and that appropriately selected combinations of supply-directed and demand-directed therapy may be more effective than either approach alone. These predictions require direct experimental and clinical testing before the framework can be considered established.

## Figures and Tables

**Table 1:** Disease-specific relationship among the initiating lesion, mitochondrial dysfunction, myelin involvement, and oxidative stress.

Disorder	Primary Affected Cell/Compartment	Initiating Molecular Defect	Mitochondrial Dysfunction	Myelin Involvement	Role of Oxidative Stress	Mechanistic Classification/Evidence
*OPA1* dominant optic atrophy	RGC soma and axon	*OPA1* dysfunction: impaired inner-membrane fusion, cristae maintenance, mtDNA stability	Primary and well supported; impaired respiration, altered dynamics and apoptotic susceptibility [19,20,35]	No evidence that demyelination initiates disease; retrolaminar myelin changes, if present, are secondary	Likely modifier downstream of mitochondrial dysfunction	Primary mitochondrial axonopathy; high evidence
*OPA3*-associated optic atrophy/Costeff syndrome	RGCs and broader nervous system in syndromic disease	*OPA3* mutation with mitochondrial metabolic dysfunction	Primary, supported by mitochondrial localization and metabolic phenotype [21]	Primary myelin pathology not established	Plausible modifier; disease-specific evidence is limited	Primary mitochondrial disorder; moderate evidence for exact mechanism
*AFG3L2*/*SPG7* optic atrophy	RGC axon and mitochondrial inner membrane	m-AAA protease dysfunction with abnormal *OPA1* processing	Primary; fragmentation and impaired respiratory function documented [36,37]	Primary myelin involvement not established	Plausible downstream amplifier	Primary mitochondrial quality-control disorder; high to moderate evidence
*TMEM126A* recessive optic atrophy	RGCs / optic nerve	Biallelic *TMEM126A* loss affecting mitochondrial function	Primary by genetic and mitochondrial localization evidence [38]	Primary myelin abnormality not established	Unresolved; likely secondary if present	Primary mitochondrial optic neuropathy; moderate evidence
Multiple sclerosis-associated optic neuritis	Optic nerve oligodendrocytes, myelin, immune cells; secondarily axons	Autoimmune inflammatory demyelination	Secondary but well supported in active lesions; mitochondrial injury contributes to axonal failure [22,23,24,25,26,27]	Initiating/core pathology	Major disease modifier and mediator of tissue injury	Primary inflammatory demyelination with secondary mitochondrial injury; high evidence
AQP4-IgG-positive NMOSD optic neuritis	Astrocytes expressing AQP4; secondary oligodendrocytes and axons	AQP4-IgG-mediated astrocyte injury with complement and inflammatory effector mechanisms	Predominantly downstream of severe inflammatory tissue injury	Secondary to astrocytopathy, not the initiating lesion [39]	Downstream mediator/amplifier of inflammatory injury	Primary astrocytopathy; high evidence for initiating lesion, moderate for mitochondrial contribution
MOG antibody-associated optic neuritis	Myelin/oligodendrocyte-associated compartment in optic nerve	MOG-IgG-associated inflammatory demyelination	Likely secondary to inflammatory and axonal stress; direct disease-specific evidence remains limited	Initiating/core pathology [40]	Plausible modifier during inflammatory injury	Primary inflammatory demyelinating disease; high evidence for myelin involvement, limited for mitochondrial mechanism

## Data Availability

No new data were created or analyzed in this study. Data sharing is not applicable to this article.

## Abbreviations

ADOA	autosomal dominant optic atrophy
AROA	autosomal recessive optic atrophy
ATP	adenosine triphosphate
CNS	central nervous system
MCT1	monocarboxylate transporter 1
mtDNA	mitochondrial DNA
OXPHOS	oxidative phosphorylation
PGC-1α	peroxisome proliferator-activated receptor gamma coactivator 1 alpha
PPAR-γ	peroxisome proliferator-activated receptor gamma
ROS	reactive oxygen species
RGC	retinal ganglion cell
AQP4	aquaporin-4
MOGAD	myelin oligodendrocyte glycoprotein antibody-associated disease
NMOSD	neuromyelitis optica spectrum disorder
VEP	visual-evoked potential
